# HTLV: It Is Time to Reach a Consensus on Its Nomenclature

**DOI:** 10.3389/fmicb.2022.896224

**Published:** 2022-04-22

**Authors:** Antonio Carlos Rosário Vallinoto, Carolina Rosadas, Luiz Fernando Almeida Machado, Graham P. Taylor, Ricardo Ishak

**Affiliations:** ^1^Laboratório de Virologia do Instituto de Ciências Biológicas da Universidade Federal do Pará, Belém, Brazil; ^2^Section of Virology, Department of Infectious Disease, Imperial College London, London, United Kingdom

**Keywords:** HTLV-1, HTLV-2, *Deltaretrovirus*, *Retroviridae*, retrovirus, taxonomy

## Introduction

Human T-lymphotropic virus 1 (HTLV-1) is an important human pathogen. A fair estimate indicates that at least 5–10 million people harbor the virus worldwide (Legrand et al., 2022), and more recently the World Health Organization (WHO) was called upon (Martin et al., 2018) to respond positively to establish adequate public health policies toward its elimination (World Health Organization, 2021). This virus is associated with a large array of diseases, including lymphoma/leukemia, neurodegenerative diseases, uveitis, infective dermatitis, Sjogren's syndrome, bronchiectasis, bronchitis and bronchiolitis, rheumatoid arthritis, arthritis, kidney and bladder infections, dermatophytosis, community acquired pneumonia, *Strongyloides* hyperinfection syndrome, tuberculosis, liver cancer, lymphoma other than adult T-cell leukemia-lymphoma and cervical cancer (Schierhout et al., 2020). Human T-lymphotropic virus 2 (HTLV-2) is still not conclusively etiologically linked to a disease although a number of disease associations have been reported particularly from the HOST study (Araújo and Hall, 2004; Orland et al., 2004; Martinez et al., 2019). Two other types, Human T-lymphotropic virus 3 (HTLV-3) and Human T-lymphotropic virus 4 (HTLV-4) have been described in a forest area in Cameroon (Wolfe et al., 2005) as result of cross-species transmission, with their occurrence restricted to this geographic area, without evidence of pathogenicity and human-to-human transmission (Duong et al., 2008; Perzova et al., 2010).

HTLV-1 and HTLV-2 share molecular properties, including the evolutionary aspect of viral and cell nucleic acid integration, silent and lifelong persistence and transmission pathways (Hall et al., 1996; Ciminale et al., 2014; Martinez et al., 2019) and are classified into the family *Retroviridae*, subfamily *Orthoretrovirinae*, genus *Deltaretrovirus*, which has four species: *Bovine Leukemia virus* and *Primate T-lymphotropic viruses* (*PTLV*) *1, 2, and 3* (ICTV—ICTV International Commitee on Taxonomy of Viruses, 2022). Within PTLV-1, 2 and 3, ICTV includes three types of human retroviruses, HTLV-1, HTLV-2, and HTLV-3, respectively. HTLV-4 remains a related but unclassified virus.

## Historical Perspective

HTLV-1 was the first described human retrovirus, isolated from a patient with a severe form of cutaneous T-cell lymphoma resembling mycosis fungoides (Poiesz et al., 1980). The first name used to describe HTLV-1 was human cutaneous T-cell lymphoma virus (HTLV; Poiesz et al., 1980) with an additional reference to its origin (HTLV_CR_). Subsequently, a second HTLV-1 isolate, HTLV-1_MB_ was described (Poiesz et al., 1981). Concurrently, investigations in Japan into the etiology of a previously known disease, Adult T-cell Leukemia/Lymphoma (ATL), resulted in the isolation of a retrovirus provisionally named Adult T-cell Leukemia Virus (ATLV) (Yoshida et al., 1982). Subsequently, HTLV-1_MB_ and ATLV were shown to be indistinguishable by a range of molecular assays (Popovic et al., 1982; Reitz et al., 1983), resulting in the suggestion to unify the viruses' names to “Human T-cell leukemia” and the disease as “adult T-cell leukemia”.

Since then, different denominations have been used: human T-cell leukemia-lymphoma virus (Gallo et al., 1982); Human T-cell lymphoma virus (Mann et al., 1983); Human T-cell leukemia virus (Clarke et al., 1983) and Human T-cell lymphotropic virus (Kühnl et al., 1985). In 1982, a new type of HTLV was isolated from a patient with an atypical form of hairy cell leukemia and named Human T-cell leukemia virus type II (Kalyanaraman et al., 1982). It is worth mentioning that by 1989, both viruses were already named as Human T-lymphotropic virus (Sodroski et al., 1984), a denomination highlighting their tropism for T lymphocytes as an important biological characteristic.

In 1991, the 5th Report of ICTV (Francki et al., 1991) listed both viruses as the species Human T-cell lymphotropic virus 1 and 2 (in the genus HTLV-BLV). In 1995, the 6th Report of ICTV (Murphy et al., 1995) kept them as species (of the genus then called BLV-HTLV), but listed both as Human T lymphotropic virus 1 and 2. In the 7th Report (van Regenmortel et al., 2000) the genus BLV-HTLV was changed to *Deltaretrovirus* and included, the species *Primate T lymphotropic virus-1* (PTLV-1, with the human type HTLV-1), PTLV-2 (with type HTLV-2) and PTLV-3 (with type HTLV-3). Since then, there were no other suggested changes in the nomenclature nor in HTLV classification.

## Discussion

Although there were several names used before and after the definition of the genus *Deltaretrovirus* and its species and types, many of the different nomenclatures were perpetuated because of the repetitive use of suggestions for the abbreviated denomination HTLV (leukemia, leukemia, lymphoproliferative, lymphotropic, etc.) with numbers either in Roman (I and II) or in Arabic (1, 2, 3, and 4) according to the preference of the authors. All of these different denominations are the cause of confusion and do not help to increase awareness both among healthcare workers or the lay community. Nowadays when search sites are essential to collate an understanding of infection and disease, important gaps appear unless all the permutations are entered as illustrated in Table 1.

**Table 1:** The impact of search systems of HTLV nomenclature/denomination on scientific literature based on PubMed.Gov.

**Nomenclature/denomination**	**Number of studies identified in PubMed**
Human T-lymphotropic virus	12,961
Human T-lymphotropic virus type 1 (or type I)	12,203
Human T-lymphotropic virus 1 (or I)	12,483
Human T-cell leukemia (or leukemia) virus	24,575
Human T-cell leukemia (or leukemia) virus type 1 (or type I)	25,837
Human T-cell leukemia (or leukemia) virus 1 (or type I)	29,820
Human T-cell lymphotropic virus	4,189
Human T-cell lymphotropic virus type 1 (or type I)	9,768
Human T-cell lymphotropic virus 1 (or I)	7,509
HTLV-1 (or HTLV-I)	18,974

A change of HTLV-1 denomination back to “human T-cell leukemia virus 1” has been proposed (Gallo et al., 2017). The arguments included “(1) Precedent throughout animal retroviruses and notably these animal retroviruses also can cause non-neoplastic disorders as well as leukemias/lymphomas, (2) Precedent as the first formal name for HTLV, (3) the fact that leukemia is the most frequent severe outcome of HTLV-1 infection, and (4) the fact that viruses commonly cause more than one disease”. The major argument is that HTLV is the most oncogenic virus known. On the other side of the argument, the proposal could not be extended to HTLV-2 (or HTLV-3 and HTLV-4) due to the lack of evidence to link them to human leukemia.

Although infection by HTLV-1 carries an enormous toll when results in leukemia/lymphoma, this is not the most common HTLV-1 associated disease diagnosed outside Japan, although underdiagnosis of ATL may occur in many countries (van Tienen et al., 2019; Rosadas et al., 2020b). In most areas of the world, the life-time risk of developing HTLV-1 associated myelopathy (HAM) is estimated to be higher than for Adult T-cell leukemia (ATL) as commonly seen for instance in Brazil (Araújo et al., 1993; Segurado et al., 1998; da Silva et al., 2013). Mild and subclinical neurological symptoms are frequently reported in patients from Brazil and were identified in up to 30% of asymptomatic subjects during 8 years of follow-up of an asymptomatic cohort in the country (Tanajura et al., 2015; Haziot et al., 2019).

A “leukemia virus”, although a highly significant denomination when describing HTLV-1, would not describe correctly such common neurological disease or the many other inflammatory conditions listed above. Furthermore, we need to consider that a “leukemia virus” would certainly increase confusion of the possible outcome among PLHTLV and healthcare professionals. Patients with asymptomatic infection or with other clinical manifestations would be led to believe that a leukemia would be their inexorable next step in this persistent infection.

Although general medical information is widely available, this is not usually true when it refers to HTLV-1 infection. There is still a lack of information about this virus. Patient's counseling is usually poor and naming the virus as “a leukemia virus” would add stress to the newly diagnosed individuals. Until today, many healthcare workers have no or only limited knowledge about this virus (Zihlmann et al., 2012). As a result, when facing a patient with “leukemia virus” they may underestimate the complexity of these individuals and the real burden of the infection, that negatively affects even those considered asymptomatic (Rosadas et al., 2020a; Schierhout et al., 2020). Indeed, in Central Australia, healthcare workers did not consider HTLV a priority (despite the extremely high infection rate among First Nation People- almost half of adult population) as they assumed that the impact of HTLV-1 on health was limited, due to rare ATL cases (Fowler and Einsiedel, 2022).

A further argument to rename HTLV to “Human T-cell Leukemia Virus” was that this had been endorsed in two distinct polls—one conducted among Global Virus Network members (16/21 voters) and the second during the 18th International Conference on Human Retrovirology (in Tokyo 2017; 78/104 voters). Although this might be taken into consideration in the process to define a virus name when there is more than one candidate for the same taxon, the policy of the ICTV is that, as far as possible, decisions on questions of taxonomy and nomenclature should reflect the majority view of the appropriate virologic constituency (Lefkowitz et al., 2018), and so far, the ICTV has not changed what was previously established since the 7th Report in 2000.

Viral taxonomy has not been always been precise and the first denomination of a virus is not always maintained. Since the establishment of the ICTV, there is a strong effort to put some order in the initial “naming” of viruses which started at the beginning of the XX century when taxonomy was quite defined and followed in botany, zoology, and microbiology. It would be highly undesirable to define two distinct taxonomic rules for both viruses (and HTLV-3, HTLV-4, and others). A fact that undeniably links HTLV-1 and HTLV-2 is that they are both lymphotropic viruses and this tropism characteristic should be the main aspect to be kept in their present denomination.

According to the International Code of Virus Classification and Nomenclature of the ICTV (https://talk.ictvonline.org/information/w/ictv-information/383/ictv-code), essential principles of virus nomenclature are: (i) to aim for stability; (ii) to avoid or reject the use of names which might cause error or confusion; (iii) to avoid the unnecessary creation of names.

Considering that: (i) the names “Human T-lymphotropic virus 1” and “Human T-lymphotropic virus 2” are comprehensive in their meaning and have been widely used, (ii) the fact that the clinical presentation goes beyond leukemia, (iii) the possibility of misleading healthcare professionals once ATL is a rare clinical manifestation, and most importantly (iv) the term “leukemia virus” is detrimental to patients as it would add stress to those diagnosed with this infection, we recommend that the name should not only be maintained but followed strictly as defined by the ICTV, which is the only institution capable of analyzing and defining viral taxonomy, since 1966. It would be desirable if editors of scientific journals could also stick to the appropriate denominations in viral taxonomy (including HTLV) to ensure a consistency in the published literature. Since the 7th Report of the ICTV in 2000, we are perpetuating an equivocal denomination. It is time to reach a consensus for the sake of consistency to refer properly to such a burden among the viruses of human medical importance.

## Author Contributions

All authors contributed to the writing and approved the final version of the manuscript.

## Funding

AV (#302935/2021-5), LM (#314209/2021-2), and RI (#312979/2018-5) are supported and acknowledge the Research Grants from the Conselho Nacional de Desenvolvimento Científico e Tecnológico—CNPq. GT was supported by the Imperial College Biomedical Research Center.

## Conflict of Interest

The authors declare that the research was conducted in the absence of any commercial or financial relationships that could be construed as a potential conflict of interest.

## Publisher's Note

All claims expressed in this article are solely those of the authors and do not necessarily represent those of their affiliated organizations, or those of the publisher, the editors and the reviewers. Any product that may be evaluated in this article, or claim that may be made by its manufacturer, is not guaranteed or endorsed by the publisher.
